# Supplementary material for: Biohydrogen production beyond the Thauer limit by precision design of artificial microbial consortia
Source: Commun Biol. 2020 Aug 14;3:443. doi: 10.1038/s42003-020-01159-x (PMC7429504; doi:10.1038/s42003-020-01159-x)
Supplement: Supplementary file 1 — Supplementary Information [file 42003_2020_1159_MOESM1_ESM.pdf]

Supplementary Information

Supplementary Tables

Supplementary Table 1. Models for cumulative pressure and μ_{mean} for *C. acetobutylicum.*

**Response**	**Cumulative pressure**					
These Rows Were Ignored for this Analysis: 8	ANOVA for Response Surface Reduced Quadratic Model			Analysis of variance table [Partial sum of squares - Type III]		
	Sum of		Mean	F	p-value	
Source	Squares	df	Square	Value	Prob > F	
Model	66.65	6	11.11	29.64	< 0.0001	significant
A-Ammonium chloride	0.2214	1	0.2214	0.5907	0.4507	
B-KH2PO4	3.63	1	3.63	9.68	0.0053	
C-Sodium acetate	0.3274	1	0.3274	0.8734	0.3606	
AB	4.58	1	4.58	12.23	0.0021	
BC	51.45	1	51.45	137.28	< 0.0001	
A^2	6.35	1	6.35	16.95	0.0005	
Residual	7.87	21	0.3748			
Lack of Fit	0.8221	2	0.4111	1.11	0.3506	not significant
Pure Error	7.05	19	0.371			
Cor Total	74.53	27				
Std. Dev.	0.6122		R-Squared	0.8944		
Mean	2.94		Adj R-Squared	0.8642		
C.V. %	20.82		Pred R-Squared	0.8124		
			Adeq Precision	15.3724		
Final Equation in Terms of Actual Factors:	Cumulative pressure	=				
	0.088345					
	0.060201	Ammonium chloride				
	0.026799	KH2PO4				
	0.124868	Sodium acetate				
	-0.000111	Ammonium chloride * KH2PO4				
	-0.001516	KH2PO4 * Sodium acetate				
	-0.000412	Ammonium chloride^2				

Response	μ mean					
These Rows Were Ignored for this Analysis: 8	ANOVA for Reduced 2FI model			Analysis of variance table [Partial sum of squares - Type III]		
	Sum of		Mean	F	p-value	
Source	Squares	df	Square	Value	Prob > F	
Model	0.0111	5	0.0022	17.25	< 0.0001	significant
A-Ammonium chloride	0.009	1	0.009	70.11	< 0.0001	
B-KH2PO4	4.19E-06	1	4.19E-06	0.0325	0.8586	
C-Sodium acetate	0	1	0	0.232	0.6348	
AB	0.0011	1	0.0011	8.84	0.007	
AC	0.0007	1	0.0007	5.78	0.0251	
Residual	0.0028	22	0.0001			
Lack of Fit	0.0002	3	0.0001	0.3807	0.768	not significant
Pure Error	0.0027	19	0.0001			
Cor Total	0.014	27				
Std. Dev.	0.0114		R-Squared	0.7968		
Mean	0.0949		Adj R-Squared	0.7506		
C.V. %	11.97		Pred R-Squared	0.686		
			Adeq Precision	12.4556		
Final Equation in Terms of Actual Factors:	μ mean	=				
	0.11866					
	-0.000369	Ammonium chloride				
	-0.00012	KH2PO4				
	0.000586	Sodium acetate				
	1.75E-06	Ammonium chloride * KH2PO4				
	-7.71E-06	Ammonium chloride * Sodium acetate				

Supplementary Table 2. Models for cumulative pressure and μ_{mean} for *E. aerogenes.*

**Response**	**Cumulative pressure**					
These Rows Were Ignored for this Analysis: 29	ANOVA for Response Surface Reduced Quadratic Model			Analysis of variance table [Partial sum of squares - Type III]		
	Sum of		Mean	F	p-value	
Source	Squares	df	Square	Value	Prob > F	
Model	53.99	5	10.8	500.23	< 0.0001	significant
A-Ammonium chloride	0.0194	1	0.0194	0.899	0.3533	
B-KH2PO4	11.17	1	11.17	517.26	< 0.0001	
C-Sodium acetate	16.14	1	16.14	747.77	< 0.0001	
BC	19.96	1	19.96	924.83	< 0.0001	
A^2	5.03	1	5.03	233.17	< 0.0001	
Residual	0.4749	22	0.0216			
Lack of Fit	0.1339	3	0.0446	2.49	0.0917	not significant
Pure Error	0.3411	19	0.018			
Cor Total	54.47	27				
Std. Dev.	0.1469		R-Squared	0.9913		
Mean	4.02		Adj R-Squared	0.9893		
C.V. %	3.65		Pred R-Squared	0.9853		
			Adeq Precision	53.1379		
Final Equation in Terms of Actual Factors:	Cumulative pressure	=				
	0.511464					
	0.047083	Ammonium chloride				
	0.025082	KH2PO4				
	0.134472	Sodium acetate				
	-0.000943	KH2PO4 * Sodium acetate				
	-0.000366	Ammonium chloride^2				

**Response**	**μ mean**					
These Rows Were Ignored for this Analysis: 8	ANOVA for Response Surface Reduced Quadratic Model			Analysis of variance table [Partial sum of squares - Type III]		
	Sum of		Mean	F	p-value	
Source	Squares	df	Square	Value	Prob > F	
Model	0.0046	5	0.0009	9.15	< 0.0001	significant
A-Ammonium chloride	9.88E-06	1	9.88E-06	0.0981	0.757	
B-KH2PO4	1.10E-03	1	1.10E-03	10.91	0.0032	
C-Sodium acetate	0.0003	1	0.0003	3.05	0.0946	
BC	0.0024	1	0.0024	23.64	< 0.0001	
AÂ^2	0.0006	1	0.0006	6.16	0.0212	
Residual	0.0022	22	0.0001			
Lack of Fit	0.0002	3	0.0001	0.7781	0.5206	not significant
Pure Error	0.002	19	0.0001			
Cor Total	0.0068	27				
Std. Dev.	0.01		R-Squared	0.6752		
Mean	0.0719		Adj R-Squared	0.6013		
C.V. %	13.96		Pred R-Squared	0.4714		
			Adeq Precision	7.6716		
Final Equation in Terms of Actual Factors:	μ mean	=				
	0.039551					
	0.00054	Ammonium chloride				
	0.000264	KH2PO4				
	0.00106	Sodium acetate				
	-1.00E-05	KH2PO4 * Sodium acetate				
	-4.07E-06	Ammonium chloride^2				

Supplementary Table 3: Global yields, substrate uptake rate and mass balance analyses of the pure cultures (*E. aerogenes* and *C. acetobutylicum*) and consortium grown on glucose and cellobiose during the cultivation.

E. aerogenes **on Glucose**

Time	Glucose uptake rate [C-mmol L^{-1} h^{-1}]	Y (L-BD/s) *	Y (Ac/s) *	Y (Form/s) *	Y (IBa/s) *	Y (Citr/s) *	Y (Et/s) *	Y (D-BD/s) *	Y (Ba/s) *	Y (x/s) *	C-balance +	DoR #
18	52.24±0.52	0.33±0.01					0.12±0.01	0.04±0.001			0.50±0.01	0.79±0.01
23.5	40.01±0.39	0.34±0.03					0.13±0.004	0.04±0.004		0.01±0.001	0.54±0.03	1.01±0.04
40.5	21.87±1.93	0.34±0.01		0.06±0.01			0.16±0.003	0.03±0.006		0.01±0.0004	0.63±0.02	1.21±0.04
45	19.68±1.57	0.34±0.01		0.07±0.01			0.15±0.004	0.03±0.006		0.01±0.0004	0.70±0.01	1.41±0.06
63	12.21±0.43	0.33±0.01		0.06±0.01			0.17±0.001	0.02±0.001			0.96±0.02	1.12±0.02

E. aerogenes **on Cellobiose**

Time	Cellobiose uptake rate [C-mmol L^{-1} h^{-1}]	Y (L-BD/s) *	Y (Ac/s) *	Y (Form/s) *	Y (IBa/s) *	Y (Citr/s) *	Y (Et/s) *	Y (D-BD/s) *	Y (Glu/s) *	Y (x/s) *	C-balance +	DoR #
17.5	1.96±6.8	0.89±0.40				0.02±0.01	0.03±0.02	0.24±0.4	0.20±0.05		0.76±0.31	0.84±0.53
22.5	14.75±4.2	0.56±0.08				0.01±0.01	0.06±0.03	0.01±0.01	0.02±0.03	0.01±0.003	0.65±0.05	0.89±0.09
40	13.37±1.29	0.35±0.02					0.14±0.04	0.05±0.03		0.02±0.002	0.72±0.03	0.79±0.03
43	12.42±1.21	0.36±0.01		0.02±0.03			0.15±0.03	0.05±0.03		0.18±0.02	1.44±0.1	1.05±0.06
61	7.77±0.76	0.34±0.01		0.05±0.03			0.20±0.04	0.03±0.02	0.05±0.09	0.04±0.004	1.05±0.03	1.12±0.03
64	7.39±0.79	0.34±0.02		0.04±0.03			0.20±0.04	0.03±0.02	0.05±0.01	0.03±0.004	1.10±0.06	1.17±0.05

C. acetobutylicum **on Glucose**

Time	Glucose uptake rate [C-mmol L^{-1} h^{-1}]	Y (N-acet/s) *	Y (Ac/s) *	Y (Form/s) *	Y (IBa/s) *	Y (Citr/s) *	Y (Et/s) *	Y (D-BD/s) *	Y (Ba/s) *	Y (x/s) *	C-balance +	DoR #
21	0.43±0.04			0.04±0.01			0.05±0.01		0.10±0.1		0.01±0.001	1.36±0.2
38.5	0.01±0.01	0.23±0.47	0.13±0.01	0.12±0.09	0.04±0.02		0.25±0.06		0.20±0.04		0.57±0.63	5.11±4.13
45	0.32±0.16	0.03±0.02	0.05±0.04	0.02±0.01	0.04±0.02		0.05±0.04		0.18±0.10	0.002	0.24±0.26	0.24±0.17
62	8.61±1.42		0.08±0.01	0.02±0.01	0.05±0.003		0.09±0.01		0.31±0.02	0.007	1.12±0.12	0.91±0.10
86.5	8.13±0.19		0.08±0.004	0.03±0.003	0.04±0.001		0.09±0.003		0.25±0.01	0.001	1.01±0.02	1.03±0.02

C. acetobutylicum on Cellobiose

Time	Cellobiose uptake rate [C-mmol L^{-1} h^{-1}]	Y (L-BD/s) *	Y (Ac/s) *	Y (Form/s) *	Y (IBa/s) *	Y (Citr/s) *	Y (Et/s) *	Y (D-BD/s) *	Y (Glu/s) *	Y (x/s) *	C-balance +	DoR #
23	0.01±0.05			0.06±0.11			0.14±0.1		0.59±0.9	0.02±0.03	0.39±0.81	1.05±0.13
28	1.74±0.30		0.08±0.02	0.01±0.01	0.28±0.07					0.001±0.00	0.37±0.08	0.25±0.06
42.5	14.48±0.22		0.08±0.03	0.02±0.00	0.28±0.01		0.06±0.03		0.01±0.01	0.01±0.00	0.85±0.02	1.15±0.03
46.5	14.02±0.11		0.08±0.02	0.02±0.00	0.28±0.01		0.06±0.01		0.01±0.00	0.13±0.07	1.02±0.02	1.20±0.02
64.5	11.94±0.04		0.08±0.01		0.26±0.02		0.06±0.01		0.01±0.00	0.01±0.00	0.66±0.02	1.10±0.02

Consortium on Glucose

Time	Glucose uptake rate [C-mmol L^{-1} h^{-1}]	Y (L-BD/s) *	Y (Ac/s) *	Y (Form/s) *	Y (IBa/s) *	Y (Citr/s) *	Y (Et/s) *	Y (D-BD/s) *	Y (Ba/s) *	Y (x/s) *	C-balance +	DoR #
16.0	0.39±0.44	2.73±1.35		1.96±0.33	3.63±0.63		5.18±2.33	2.64±1.86		0.31±0.29	0.48±0.32	0.49±0.18
20.0	1.27±0.81	0.03±0.19	0.12±0.04			0.08±0.07			0.25±0.15	0.01±0.00	0.50±0.01	0.16±0.04
34.5	21.58±3.85	0.35±0.06		0.04±0.01			0.11±0.04	0.02±0.01	0.05±0.07	0.01±0.00	0.74±0.00	1.08±0.08
39.5	21.77±0.64	0.31±0.10		0.04±0.01			0.11±0.05	0.02±0.01	0.06±0.08	0.04±0.02	1.27±0.04	1.25±0.17
53.0	17.25±0.05	0.27±0.07		0.04±0.01			0.10±0.05	0.01±0.01	0.09±0.07	0.05±0.02	0.93±0.02	1.13±0.14

Consortium on Cellobiose

Time	Cellobiose uptake rate [C-mmol L^{-1} h^{-1}]	Y (L-BD/s) *	Y (Ac/s) *	Y (Form/s) *	Y (IBa/s) *	Y (Citr/s) *	Y (Et/s) *	Y (D-BD/s) *	Y (Ba/s) *	Y (x/s) *	C-balance +	DoR #
23	0.41±0.24	0.22±0.2	0.45±0.31	0.56±0.51		0.17±0.20			1.02±0.84		0.92±0.2	0.59±0.31
28	2.30±0.67	0.10±0.08	0.20±0.07	0.03±0.03	0.01±0.00	0.01±0.04	0.02±0.01		0.27±0.11	0.01±0.01	0.58±0.15	0.57±0.15
42.5	13.88±1.42	0.02±0.02	0.09±0.01	0.03±0.00	0.01±0.00		0.05±0.00		0.28±0.02	0.01±0.00	0.72±0.05	0.75±0.06
46.5	13.74±0.23	0.02±0.02	0.09±0.01	0.02±0.00	0.01±0.00		0.05±0.00		0.27±0.03	0.06±0.03	1.00±0.08	1.02±0.09
64.5	12.05±1.35	0.01±0.01	0.08±0.01	0.01±0.00	0.03±0.01		0.05±0.00		0.21±0.04	0.01±0.00	0.47±0.05	1.24±0.14

*Y; Yield of product (L-Butanediol (L-BD), Acetic acid (Ac), Formic acid (Form), Isobutyric acid (IBa), Citric acid (Citr) Ethanol (Et), D-Butanediol (D-BD), Butyric acid (Ba), x (biomass), CO_2, H_2, N-acetyl-D-glucosamine (N-acet)) C-mol per C-mol substrate consumed

+Carbon balance

#Degree of reduction balance

Supplementary Table 4. Reactions for each organism and consortium on glucose and cellobiose at the time point where maximum $Y_{(H2/S)}$ was produced.

Glucose

E. aerogenes

$C_6H_{12}O_6 + 0.39C_2H_4O_2 + 0.27C_2H_5OH + 0.06C_3H_6O_3 + H_2O \rightarrow 1.67CH_2O_2 + 0.81C_4H_{10}O_2 + 1.38CO_2 + 0.84H_2$ + x + unknown-product
Glucose+ 0.39acetic acid + 0.27ethanol + 0.06lactic acid + $H_2O \rightarrow$ 1.67formic acid + 0.81butanediol + 1.38CO_2 + 0.84H_2 + biomass + unknown-product

C. acetobutylicum

$C_6H_{12}O_6 + 2H_2O \rightarrow 0.31CH_2O_2 + 0.27C_2H_4O_2 + 0.25C_2H_5OH + 0.20C_4H_8O_2 + 2.76CO_2 + 1.98H_2$ + x + unknown-product
Glucose + $2H_2O \rightarrow$ 0.31formic acid + 0.27acetic acid + 0.25ethanol + 0.20butyric acid + 2.76CO_2 + 1.98H_2 + biomass + unknown-product

Consortium

$C_6H_{12}O_6 + 0.95C_4H_{10}O_2 + 0.07C_2H_5OH + 0.04C_6H_8O_7 + 4H_2O \rightarrow 0.53CH_2O_2 + 0.40C_2H_4O_2 + 0.99C_4H_8O_2 + 4.04CO_2 + 5.58H_2$ + x + unknown-product
Glucose + 0.95butanediol + 0.07ethanol + 0.04citric acid + $4H_2O \rightarrow$ 0.53formic acid + 0.40acetic acid + 0.99butyric acid + 4.04CO_2 + 5.58H_2 + biomass + unknown-product

Cellobiose

E. aerogenes

$C_{12}H_{22}O_{11} + 0.26C_2H_4O_2 + 0.06C_6H_{12}O_6 \rightarrow 0.2CH_2O_2 + 0.37C_4H_{10}O_2 + 1.59C_2H_5OH + 0.96CO_2 + 0.48H_2$ + x + unknown-product
Cellobiose + 0.26acetic acid + 0.06glucose $\rightarrow$ 0.2formic acid + 0.37butanediol + 1.59ethanol + 0.96CO_2 + 0.48H_2 + biomass + unknown-product

C. acetobutylicum

$C_{12}H_{22}O_{11} + 0.03C_4H_{10}O_2 + 0.19C_6H_{12}O_6 + 2H_2O \rightarrow 0.83C_2H_4O_2 + 0.56C_2H_5OH + 1.31C_4H_8O_2 + 4.56CO_2 + 5.16H_2$ + x + unknown-product
Cellobiose + 0.03butanediol+ 0.19glucose + $2H_2O \rightarrow$ 0.83acetic acid + 0.56ethanol + 1.31butyric acid + 4.56CO_2 + 5.16H_2 + biomass + unknown-product

Consortium

$C_{12}H_{22}O_{11} + 0.03CH_2O_2 + 8H_2O \rightarrow 0.37C_2H_4O_2 + 0.04C_4H_{10}O_2 + 0.22C_2H_5OH + 0.25C_4H_8O_2 + 0.01C_6H_{12}O_6 + 8.64CO_2 + 8.76H_2$ + x + unknown-product
Cellobiose + 0.03formic acid + $8H_2O \rightarrow$+ 0.37acetic acid + 0.04butanediol + 0.22ethanol + 0.25butyric acid + 0.01glucose + 8.64CO_2 + 8.76H_2 + biomass + unknown-product

Supplementary Table 5. Shannon index (H), species richness (S) and evenness (E_H) values of consortium on glucose and cellobiose.

Consortium on Glucose

Time	H	S	E_H
**0**	0.00	2	0.00
**16**	0.75	2	0.75
**20**	0.94	2	0.94
**34.5**	0.14	2	0.14
**39.5**	0.51	2	0.51
**53**	0.89	2	0.89

Consortium on Cellobiose

Time	H	S	E_H
**0**	0.00	2	0.00
**17**	0.26	2	0.26
**22.5**	0.86	2	0.86
**39.5**	0.93	2	0.93
**42.5**	0.79	2	0.79
**59.5**	0.44	2	0.44

Supplementary Table 6. qPCR primers for quantification of *C. acetobutylicum* and *E. aerogenes*

Target	Primer	DNA Sequence (5'-3')	Gene	Product Length (nt)
C. acetobutylicum	C_F<br>C_R	TGG CAC AGT CAG TCG<br>GCT ACC GCG TGA TGC<br>ACC TAA CCC AGC	ABC transporter (permease) (AEI33449.1)	108
E. aerogenes	E_F<br>E_R	GCG TTG TGG GGT TGC<br>ACG ATTGG CGC GCG<br>AGC ACA TTT TC	Cation diffusion facilitator family transporter (AEG98846.1)	106

Supplementary Table 7. FISH probes for the *in situ* monitoring of *E. aerogenes, C. acetobutylicum* and the consortium

Target	Probe	DNA Sequence (5'-3')	Fluorophore	Reference
C. acetobutylicum<br>*E. aerogenes*	EUB338	GCTGCCTCCCGTAGGAGT	Cy3	[1]
E. aerogenes	GAM42a	GCCTTCCCACATCGTTT	Fluos	[2]

Supplementary Figures

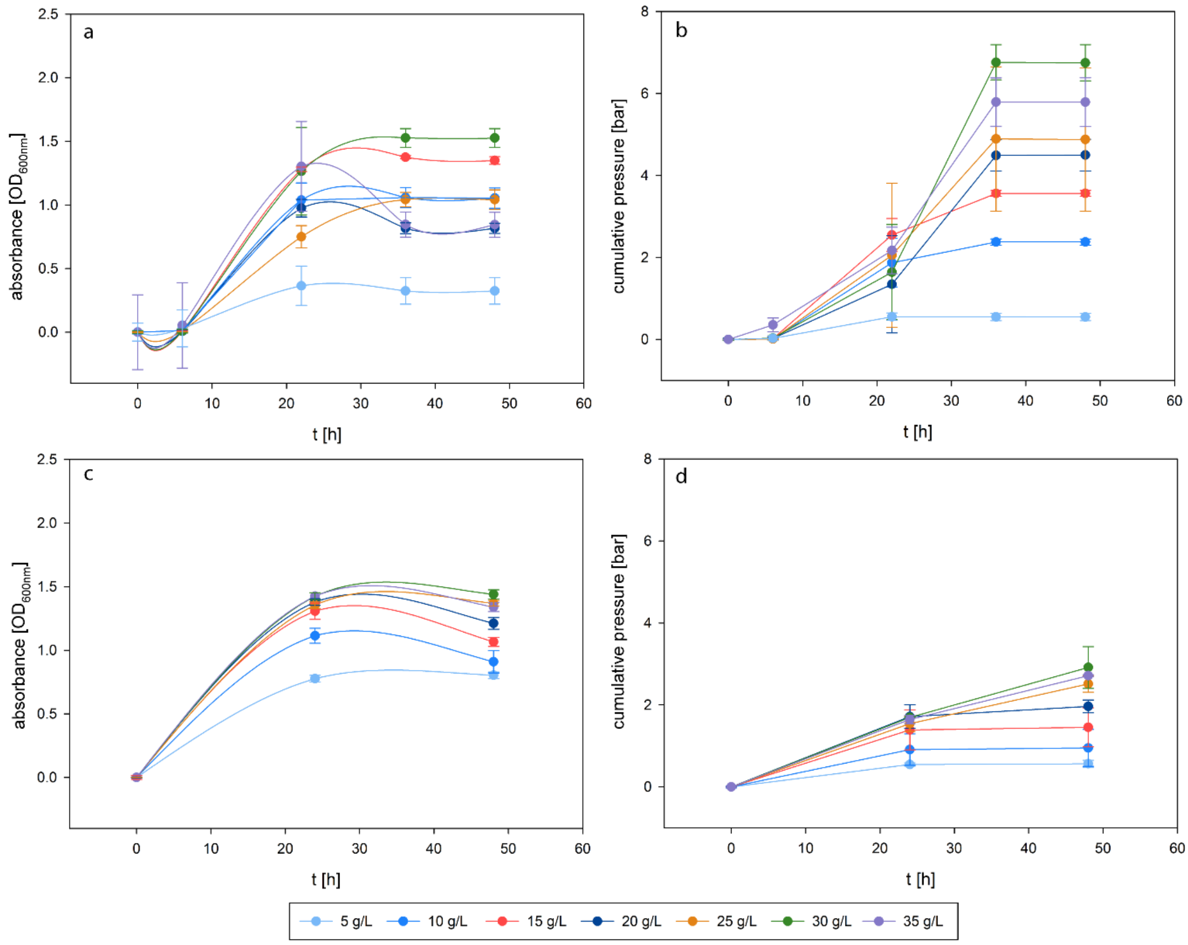

Supplementary Fig. 1. Optical density and cumulative pressure measurements of *C. acetobutylicum* on Clostridia-specific medium (a-b) and *E. aerogenes* on Enterobacter-specific medium (c-d) with different glucose concentrations ranging from 5 to 35 g L^{-1}. Experiments were replicated three times (three different sets, N=3), and each set contained quadruplicate (n=4).

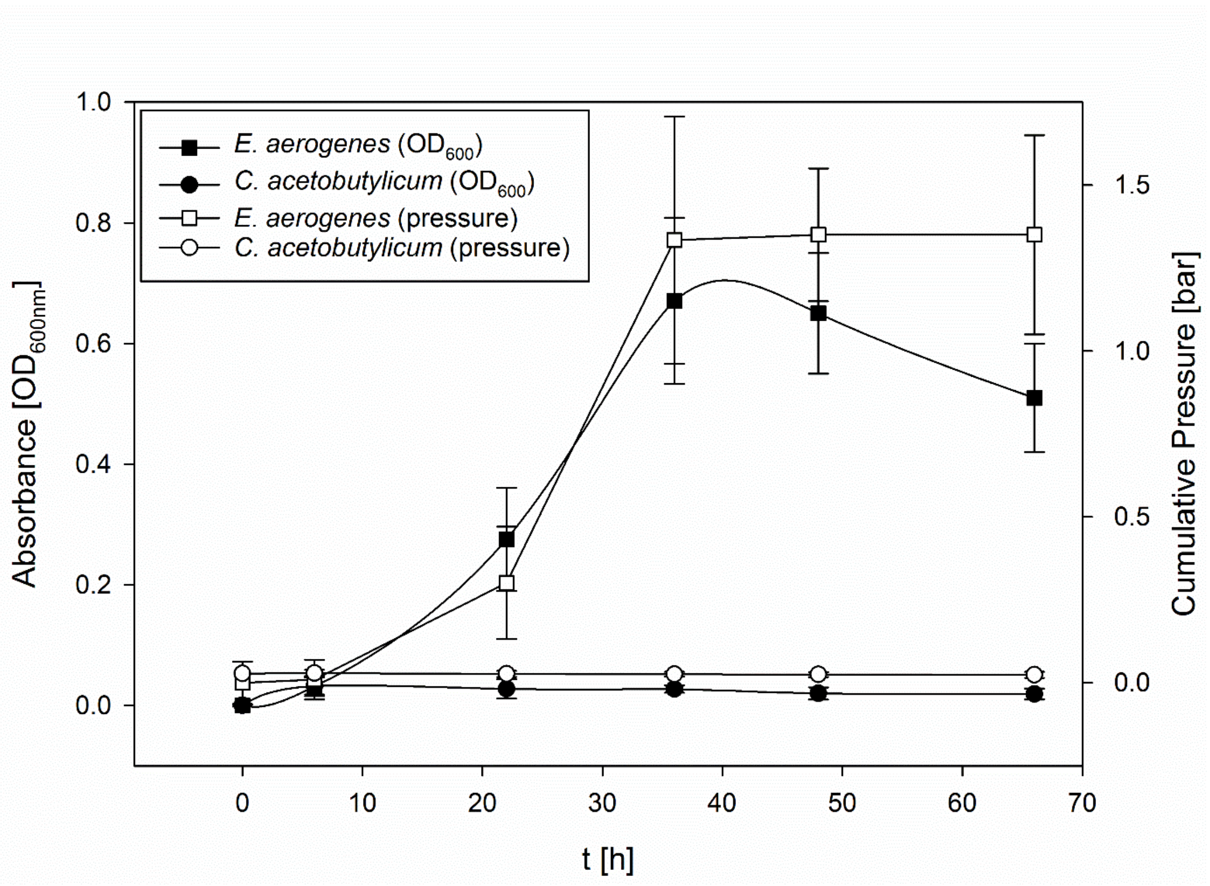

Supplementary Fig. 2. Optical density and cumulative pressure measurements of *C. acetobutylicum* on Enterobacter-specific medium and *E. aerogenes* on Clostridia-specific medium. *C. acetobutylicum* did not grow in Enterobacter-specific medium.

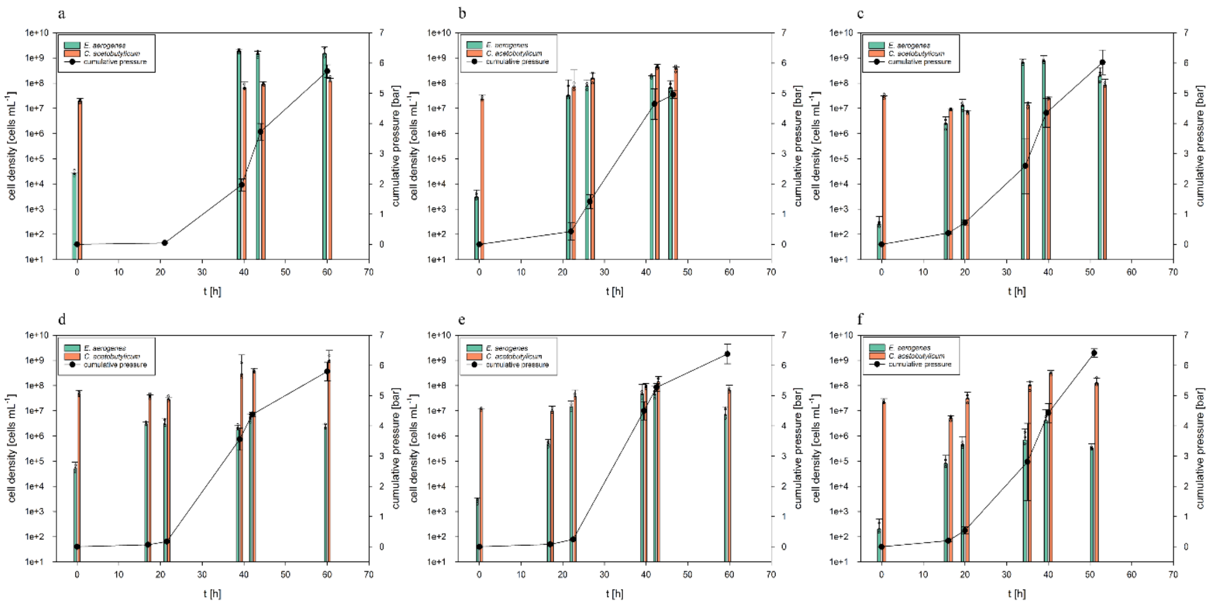

Supplementary Fig. 3. Growth and gas production of consortia inoculated with different initial cell densities of *E. aerogenes* : *C. acetobutylicum* ratios of 1:100 (a-d), 1:1,000 (b-e) and 1:10,000 (c-f) on glucose and cellobiose, respectively.

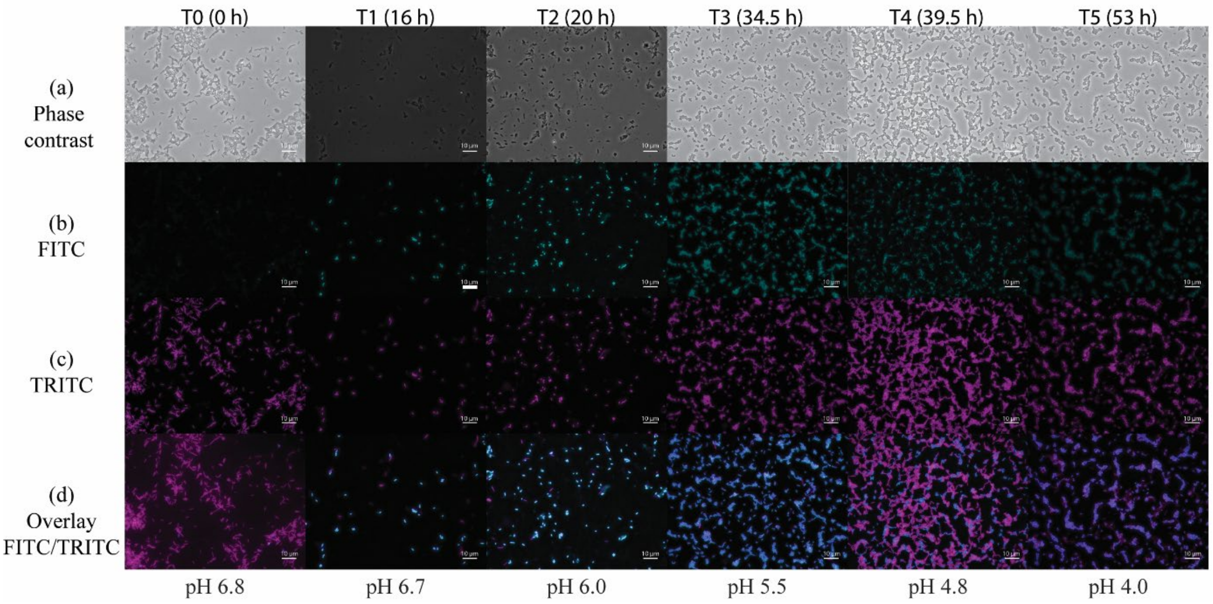

Supplementary Fig. 4. Fluorescent in-situ hybridization (FISH) analysis of the consortium on glucose during cultivation. Samples (T0 to T5) taken during cultivation were visualised for detecting the growth pattern of *E. aerogenes* and *C. acetobutylicum* in the consortium. The phase contrast images (a), FITC filter set images (representing *E. aerogenes* (green) labelled with GAM42a probe) (b), TRITC filter set images (representing both *E. aerogenes* and *C. acetobutylicum* (pink) hybridised with EUB338 probe) (c) and images from overlay of FITC/TRITC filter sets (after overlay *E. aerogenes* appears blue, *C. acetobutylicum* appears pink) (d) are represented. The scale bar is 10 µm.

References

1. Amann, R. I. *et al.* Combination of 16S rRNA-targeted oligonucleotide probes with flow cytometry for analyzing mixed microbial populations. *Appl. Environ. Microbiol.* **56**, 1919–1925 (1990).
2. Manz, W., Amann, R., Ludwig, W., Wagner, M. & Schleifer, K.-H. Phylogenetic Oligodeoxynucleotide Probes for the Major Subclasses of Proteobacteria: Problems and Solutions. *Syst. Appl. Microbiol.* **15**, 593–600 (1992).
