# Supplementary material for: Biohydrogen production beyond the Thauer limit by precision design of artificial microbial consortia
Source: Commun Biol. 2020 Aug 14;3:443. doi: 10.1038/s42003-020-01159-x (PMC7429504; doi:10.1038/s42003-020-01159-x)
Supplement: Supplementary file 4 — Description of Additional Supplementary Files [file 42003_2020_1159_MOESM4_ESM.pdf]

Description of Additional Supplementary Files

File Name: **Supplementary Data 1**

Description: Previous consortia studies with respect to dark fermentative H2 production and their main parameters.
